# Implementation of a Pharmacy Follow-Up Program for Dispensed Opioid Medications

**DOI:** 10.3390/ijerph20176628

**Published:** 2023-08-23

**Authors:** Elizabeth Skoy, Oliver Frenzel, Haley Pajunen, Heidi Eukel

**Affiliations:** Department of Pharmacy Practice, School of Pharmacy, North Dakota State University, Dept 2660, Fargo, ND 58108, USA; oliver.frenzel@ndsu.edu (O.F.); haley.pajunen@ndsu.edu (H.P.); heidi.eukel@ndsu.edu (H.E.)

**Keywords:** pharmacy technician, opioid medications, follow-up, medication disposal, medication safety

## Abstract

Background: There have been multiple reported pharmacy initiatives to reduce opioid misuse and accidental overdose to address our nation’s public health crisis. To date, there has not been a description in the literature of a community pharmacy follow-up initiative for dispensed opioids. Methods: A follow-up program was designed and implemented in community pharmacies as part of a previously developed opioid overdose and misuse prevention program (ONE Program). Five to twelve days after the dispensing of an opioid, pharmacy technicians called the patient to follow up on opioid safety topics. Pharmacy technicians used a questionnaire to inquire about medication disposal plans, if the patient was taking the medication more than prescribed, medication side effects, and if the patient needed a pharmacist consultation. The results from that questionnaire were documented. Results: During the first 18 months of the follow-up program, 1789 phone calls were completed. Of those contacted, 40% were still using their opioid medication, and over 10% were experiencing side effects which triggered a pharmacist consult. Patients were reminded of proper medication disposal methods, and most patients (78%) desired to dispose of unused medication at the pharmacy medication disposal box. Conclusions: Follow-up phone calls post-opioid medication dispensing were shown to add value to a previously established opioid misuse and accidental overdose prevention program and allowed for the fulfillment of the Pharmacist Patient Care Process.

## 1. Introduction

The opioid epidemic has been an ongoing United States public health crisis for the past decade and it is expected to continue to be an issue for years to come [1]. In the year 2020, approximately 9.5 million people misused opioids [2]. Opioid misuse is a multifactorial issue, but it is clear that improper disposal of unused medication is a contributor to misuse, since 47.2% of those that report misusing opioids obtain them from a friend or relative [2]. Patients often do not know or understand the risks of retaining opioids and keep them on hand in case of future need, because they are unaware of disposal options, and find the medication valuable [3,4]. In addition to disposal practices, improper patient monitoring and follow-up can also contribute to opioid misuse [2,5,6].

The pharmacy profession has demonstrated its ability to help combat the opioid epidemic [7,8,9,10]. As pharmacists continue to transition from the exclusive role of a dispenser to more of a provider role, they are being challenged to take greater ownership of patient health outcomes and foster connectivity through public health service efforts. Research has revealed that accessibility of community pharmacists- and pharmacy-centered patient care programs including initiatives targeting opioid safety and disposal can elevate patient engagement and community wellbeing [11,12,13,14,15].

One such program is titled the ONE (Opioid and Naloxone Education) Program, and has been operating within community pharmacies in North Dakota since 2018 [16,17,18]. The program has provided opioid misuse and accidental overdose risk assessment screening for over 14,000 patients. Safe opioid use and disposal practices continue to be at the forefront of this program. The program was designed and has strived to follow the Pharmacists’ Patient Care Process (PPCP). The PPCP is a tool developed by the Joint Commission of Pharmacy Practitioners to standardize the patient care process across pharmacy settings. The PPCP includes five steps: collect, assess, plan, implement, and follow-up [19]. The follow-up component is important but often overlooked, and includes patient monitoring and evaluation [20].

Follow-up is essential during opioid treatment because of the high risk of misuse and accidental overdose [21,22]. The Journal of American Pharmacists Association published a mixed methods study that highlighted strategies for follow-up in the community pharmacy setting [17]. Results showed that delegating the task of patient follow-up to a nonpharmacist staff, such as a pharmacy technician, may increase workflow efficiency. Thus, community pharmacists could focus on providing patient care according to the PPCP [23].

In recent years, there has been a large expansion in the role of pharmacy technicians. In the community and ambulatory care settings, advanced pharmacy technician roles include immunizations, medication billing and reimbursement, 340 B program support, and controlled substance diversion program oversight [24]. In addition, diversifying the pharmacy technician role and shifting away from traditional product dispensing positively impacts pharmacy technician job satisfaction, increases self-actualization, and provides meaningful activities that benefit work performance [25].

Since follow-up is deemed essential to patient care after the receipt of opioid medication, and there is a demonstrated need for disposal practice education, it seems fitting to have the community pharmacy take on this role. However, to date there has not been any description in the literature of a community pharmacy follow-up initiative for dispensed opioids. The objective of this manuscript is to describe the implementation and results of a pharmacy technician follow-up initiative for individuals prescribed an opioid medication.

## 2. Materials and Methods

The ONE Program is an established program that places community pharmacists at the forefront of prevention of opioid misuse and accidental overdose. Reports of the program and its success have been previously reported in the literature [17,18,26,27]. In summary, community pharmacies enrolled in the ONE Program objectively screen each patient for risk of opioid misuse and accidental overdose prior to the dispensing of an opioid medication. Based on individual patient risk, the pharmacist then provides patient-specific interventions such as counseling on opioid use disorder, prescribing and dispensing naloxone, discussing medication disposal, and contacting the patient’s provider if needed. The pharmacist then documents the patient screening results and provided interventions in an electronic reporting system. For every patient screened, the pharmacist is reimbursed USD 20 through a grant provided by the North Dakota Department of Health and Human Services.

**Figure 1:**
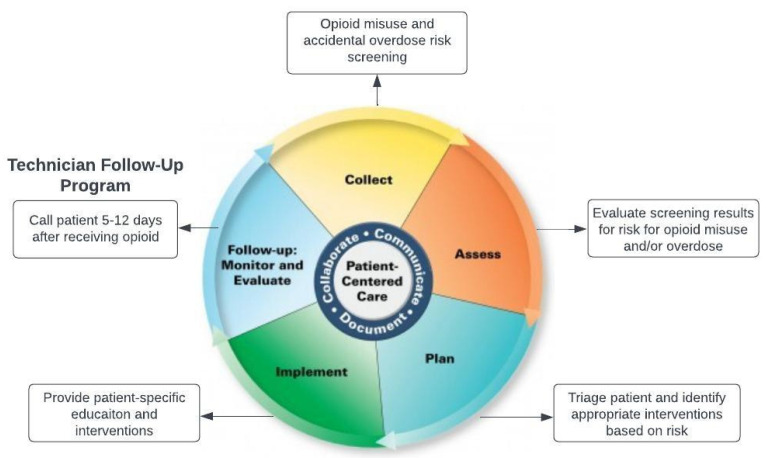
Pharmacist patient care process and the ONE Program.

The program closely follows the PPCP (Figure 1). The pharmacist collects information from the patient, assesses their risk of opioid misuse and accidental overdose, creates a plan to prevent these effects, and then implements interventions. Over time, it became apparent that one component of the PPCP was missing. Originally, pharmacists were providing risk-specific interventions with high fidelity but patient follow-up was not incorporated into the program. In addition, although recommendations for disposal have been an integral part of the ONE Program from its inception, it was uncertain as to which disposal method was preferred by patients.

**Figure 2:**
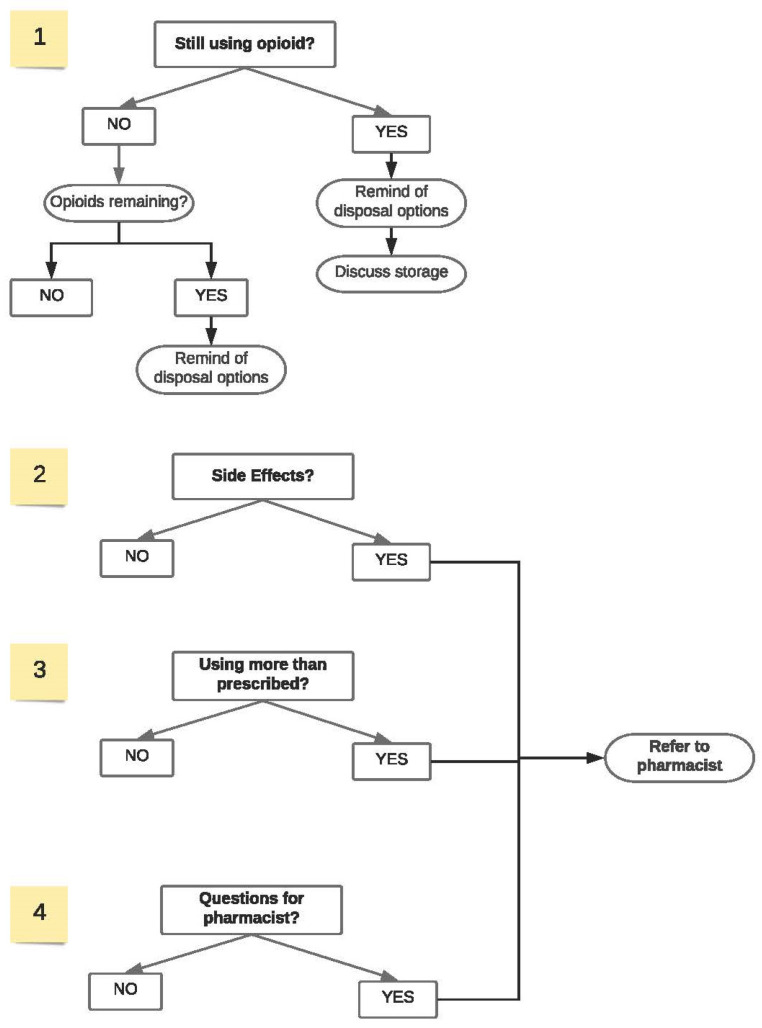
Technician follow-up process.

To address these issues, recognizing that follow-up initiatives can be less burdensome in workflow with the assistance of pharmacy technicians, the pharmacy technician follow-up initiative was created. Each follow-up phone call was generated as a task in an electronic reporting system 5–12 days after an initial ONE Program screening. This timeframe was chosen based on the average length of treatment for acute conditions typically treated with opioid medication. Within the timeframe window, a pharmacy technician was to call the patient and ask a series of questions related to opioid medication use, side effects, and disposal (Figure 2). Pharmacy technicians then recorded the patient response in the electronic reporting system. For every follow-up phone call completed and recorded, the pharmacy was reimbursed an additional USD 10 to the original USD for 20 ONE Program screening.

Prior to the official launch of the follow-up initiative, authors collaborated with a pharmacy technician to gather input and feedback to the follow-up process and questionnaire (Figure 2). This collaboration was to ensure the questionnaire would be adopted into a pharmacy technicians’ workflow and would be easy to understand. In addition, to ensure program fidelity, an optional one-hour, on-demand continuing education program was provided free of charge to pharmacy technicians. The education provided an overview of the ONE Program, the importance of opioid medication follow-up, and the follow-up initiative process.

Pharmacies participating in the program were provided free options for medication disposal from the ONE Program, local public health units, and the State Board of Pharmacy. These options included Deterra bags, DisposeRx packets, and medication disposal kiosks in the physical space of the pharmacy.

## 3. Results

The first 18 months of the follow-up program initiative resulted in 1789 completed phone calls (Table 1). Of patients still using an opioid analgesic, 719 (40.2%) were still using the medication and 99.6% of these patients were provided with a reminder of proper medication storage. A total of 706 (39.5%) of patients were reminded of proper disposal options available and were asked their preferred method to dispose of unused opioids.

Approximately one in every 10 patients reported experiencing side effects from the opioid medication (10.2%) and 8.2% of patients were identified by the pharmacy staff as requiring additional pharmacist consultation to address concerns relating to the opioid prescription. In addition, 1.3% of patients reported misusing the opioid prescription by taking more than prescribed and 1.8% of patients requested the pharmacist to address additional questions relating to the medication, side effects, or therapy.

Table 2 provides preferred patient disposal methods. The majority (78.6%) preferred to return the medication to the pharmacy, with the second most frequent response (18.6%) choosing to use an approved at-home disposal method such as coffee grounds or cat litter.

## 4. Discussion

Community pharmacies have risen as important facilitators in delivering services and promotion of preventative health initiatives with a transition from supplying medications to providing patient-centered services [28]. The Joint Commission of Pharmacy Practitioners developed the PPCP to establish pharmacists’ consistent delivery of care across various practice settings. The PPCP aligns with the overall importance of continuity of care (COC), which consists of individual patient care and care provided over time [19,29]. Incorporation of the COC has suggested improvements in patient outcomes and reduced inappropriate medication behaviors [29]. The ONE Program follow-up initiative is founded in these principles by providing individualized education to patients receiving prescription opioid medication and connecting with patients over time to reinforce medication safety and proper disposal practices.

National data indicate that of individuals reporting opioid misuse, nearly 50% had misused a prescription opioid obtained from a friend or family member [2]. In addition, studies have identified that a high proportion of patients using prescription opioids for acute pain (73–77%) store their opioid medications in unlocked areas [30]. The follow-up initiative results indicated that 40.2% of patients contacted by the pharmacy staff reported continued use of the opioid medication, with nearly all patients receiving information on proper storage of the medication. This portion of the follow-up education focusing on proper storage practices may cue individuals to adopt secure storage and proper disposal of opioid medications to deter inappropriate medication utilization by individuals not prescribed the opioids.

Appropriate opioid disposal is another avenue in which opioid injuries, diversion, and misuse can be mitigated by reducing stockpiling and future nonmedical opioid use by patients. Studies have reported that a minority of patients (4–30%) intend to dispose of unused opioids after pain management is complete [31,32]. Educating patients on proper disposal practices and providing informational support in how/where to dispose of unused opioids has the potential to reduce opioids in households and potential misuse by nonprescribed individuals. Our study demonstrated only a 33% completion rate for reminding patients of proper disposal options; however, only 40% of patients were still taking their medication at the time of follow-up.

Our data suggest that patients prefer to dispose of their unused medication by returning them to the pharmacy disposal box. This aligns with previous research stating that patients prefer takeback programs at the pharmacy for medication disposal over in-home disposal options [33]. North Dakota is fortunate to have a statewide medication disposal initiative. Our findings strengthen the need for more public health initiatives to make free pharmacy medication disposal options more widely available.

The ONE Program follow-up initiative provides a comprehensive method in coupling medication disposal and storage education with availability for pharmacist consultation if the patient is requiring additional support to address current medication misuse and/or side effects from the opioid medication. The ONE follow-up program results indicated that 1 in every 10 patients was currently experiencing adverse side effects at the time of the follow-up call. This unique opportunity to address specific patient concerns after initial medication dispensing has the potential to positively influence patient quality of life and reduce negative outcomes during prescription opioid utilization.

Although the follow-up program was designed to be completed by pharmacy technicians, it did not prevent pharmacies from utilizing pharmacy student interns or pharmacists instead of pharmacy technicians to complete the calls. Recognizing that pharmacies vary greatly in workforce, flexibility of the program was important for uptake and sustainability. The outcomes of the screening are objective and not affected by the credentials of who makes the follow-up calls.

There are some limitations to our study. First, this study took place within one state with broad access to medication disposal boxes within community pharmacies. These boxes are provided free of charge to pharmacies by the State Board of Pharmacy and incur no cost to the pharmacies themselves. In addition, pharmacies were provided Deterra and DisposeRx packets through grant funding at no cost to distribute to patients prescribed an opioid medication. Not all states and pharmacies will have access to such a wide range of free disposal options to offer their patients. Another limitation is that our results were based on patients self-reporting, and we are unable to confirm that patients disposed of their medication as reported. Confirmation of disposal can be challenging and labor-intensive, but future research could investigate self-reporting versus actual disposal. Additionally, phone calls being unanswered by patients decreased our response rate. This can partially be attributed to a cultural shift in communication preferences such as text messaging or email to traditional phone calls. Future research could include alternative means to contact patients for follow-up such as a text message or survey response. The ONE Program is working to provide additional follow-up communication methods for the future. Future research could also include collecting patient demographic information and medication details to identify possible trends or patterns among certain demographic groups in terms of medication usage or reported side effects. Finally, due to the novelty of this research, authors were unable to find comparable research within and/or outside the United States. Future research could include expanding the follow-up program to other areas of the United States and internationally to compare results.

## 5. Conclusions

The follow-up program described in this study adds to a pre-established ONE Program to embrace and fulfill the PPCP as applied to patients prescribed an opioid medication. The provision of educational support for disposal and storage practices, early identification of opioid misuse behavior, and instrumental support to patients experiencing side effects during opioid prescription utilization are paramount in the mitigation strategies for addressing the opioid crisis. Opioid prescription patient follow-up is an additional strategy to the multifaceted approach required in addressing opioid use disorder and accidental opioid overdoses.

## Figures and Tables

**Table 1:** Follow-up questionnaire outcomes based on completed calls (*n* = 1789).

Questionnaire Outcomes	Number	Percentage
Patients still taking opioid medication	719	40.2
Patients reminded of safe medication storage	716	99.6
Patients reminded of proper disposal options	706	33.95
Patients experiencing side effects from opioid	182	10.2
Patients taking more medication than prescribed	24	1.3
Patients have questions for the pharmacist	32	1.8
Patients referred to the pharmacist for added counseling	147	8.2

**Table 2:** Patient preferred disposal option based on those reporting (*n* = 616).

Disposal Preference	Number	Percentage
Pharmacy disposal box	555	78.6
Deterra bag	54	7.6
DisposeRx	9	1.5
Either Deterra or pharmacy disposal box	21	3.4
In-home disposal (cat litter/coffee grounds)	24	1.3

## Data Availability

Data is unavailable due to privacy or ethical restrictions.
